# Omics Landscape in Disease Biomarkers Discovery

**DOI:** 10.1155/2016/4068252

**Published:** 2016-10-24

**Authors:** Monica Neagu, Caterina Longo, Simone Ribero

**Affiliations:** ^1^Victor Babes National Institute of Pathology, Bucharest, Romania; ^2^Faculty of Biology, University of Bucharest, Bucharest, Romania; ^3^Skin Cancer Unit, Arcispedale Santa Maria Nuova-IRCCS, Reggio Emilia, Italy; ^4^University of Turin, Turin, Italy

Biomarker discovery has an accelerated pace is taking advantage of ever growing omics technologies. In the last years we are facing the development of a myriad of technologies searching for the “golden biomarker.” The omics family is constantly adding a new member, such as genomics, proteomics, peptidomics, newer ones, epigenomics, transcriptomics, metabolomics, lipidomics, pharmacogenomics, interactomics, and chronemics, just mentioning a few [1].

In the biomarker development, approaches are multidisciplinary, from basic to translational and clinical research. As biological processes are complex, for example, tumorigenesis, multitechnological approaches are crucial for protein/gene biomarkers discovery. Biomarker discovery goes through laborious individual phases, such as biomarker candidate identification, verification, and validation. In all these phases, various omics technologies take the lead. Depending on the development stage, when characterizing thousands to tens of thousands of proteins, the key technological players are mass spectrometry, multiplexed assays, and protein microarrays. For genomic studies, technological platforms of gene microarrays, next-generation sequencing, and mass spectrometry generate tumor-associated genes and potential biomarkers. Complementary omics approaches intended for biomarker candidates selection should focus on multiple proteomic/genomic/metabolomic targets. These multifaceted targets could better explain complex disease mechanisms [2].

The special issue is governed by three tumor pathologies, skin cancer, lung cancer, and colorectal cancer, while precancerous lesions like ulcerative colitis or particular inflammatory conditions like keratoconus, kidney disease, or microRNAs in pathological pregnancy are also tackled.

Hence from the large group of skin cancers papers, M. Lupu et al. review the genomic and the proteomic profile of basal cell carcinoma, the world's leading skin cancer. The paper analyzes the gene expression and proteomic profiling of both tumor cells and tumoral microenvironment. These patterns can represent novel predictive and prognostic biomarkers in BCC.

The second most frequent skin cancer squamous cells carcinoma is reviewed by V. Voiculescu et al. where environmental factor ultraviolet light, chronic scarring/inflammation, exposure to chemical compounds, and immune suppression can favour tumorigenesis. The paper shows that malignant keratinocyte proteome analysis is the pool for novel biomarker discovery future to be used in early detection, risk assessment, tumor monitoring, and development of targeted therapeutic strategies.

Another skin related paper focuses on melanoma development and G. Turcu et al. revised an interesting new possible biomarker, carcinoembryonic antigen cell adhesion molecule 1 (CEACAM1) with a definite role in melanoma metastasis. Knowing that melanoma progression is associated with cell adhesion molecules dysregulation, this molecule can further stratify high risk patients identifying subgroups that require close follow-up or more aggressive therapy.

Lung cancer was approached in two papers of this special issue. A. Quintanal-Villalonga et al. reviewed tyrosine kinase receptor with its therapeutical implications in lung cancer showing that in the “omics” landscape tyrosine kinase receptors gained their role in the different histological types of lung cancer. This receptor is highly involved in specific antitumoral therapies.

In the same field of lung cancer, A. Marrugal et al. reviewed the cytokines pattern due to their involvement in the inflammatory processes that can trigger and sustain tumorigenesis. Different proteomic techniques are presented in the continuous quest for biomarkers discovery in lung cancer. Within the future biomarker candidates, several cytokines are presented as important players in lung cancer that can stand for future therapy targets.

Colorectal cancer was another important subject presented in this special issue where J. Wei et al. presented their findings regarding hypermethylated markers. They have mapped DNA methylation profiling in colorectal cancer tissues. Over 1500 differentially methylated regions were found that proved to be different in colorectal cancer compared to normal tissue. Among these differentially methylated regions two genes (ADD2 and AKR1B1) are presented as potential screening markers of colorectal cancer.

Another original research paper within this issue is the one presented by C. Popp et al. regarding the specific expression profile of p53 and p21 in large bowel mucosa. The expression of p53 and p21 can be considered biomarker of inflammatory-related carcinogenesis in ulcerative colitis as a disease that slightly increases the risk of colorectal cancer in patients. The paper presents a one-year prospective observational study that shows results for future use of p53 as the most valuable tissue biomarker in ulcerative colitis surveillance, identifying higher risk for dysplasia patients.

Similarly, in the paper of C. Ionescu et al., inflammatory biomarkers for keratoconus are revised. Keratoconus, a degenerative disorder, results in decreased vision and irreversible tissue scarring and still has an unclear pathogenesis. Combination of inflammatory patterns and genetic and environmental factors are incriminated and studied in this type of special pathology. This paper revises biomarkers of inflammation and their signaling pathways that can indicate the etiopathogeny of this disease and that furnish new therapeutic possibilities.

Another pathology, chronic kidney disease-related biomarkers are presented by the original work of S. Mihai et al. Within the paper a panel of proteomic biomarkers, comprising inflammation markers (IL-6 and TNF-*α*) and mineral and bone disorder biomarkers (OPG, OPN, OCN, FGF-23, and Fetuin-A), was found to detect early chronic kidney disease.

Although a normal physiological condition, pregnancy can have a wide range of pathological conditions. D. Cretoiu et al. revise circulating microRNAs as potential biomarkers in pregnancy and show the significance of these circulating molecular markers from the implantation period to preeclampsia and their involvement in pathological processes like recurrent abortion and ectopic pregnancy.
